# Cost-effectiveness of specialist eating disorders services for children and adolescents with anorexia nervosa: a national surveillance study

**DOI:** 10.1186/s40337-021-00433-5

**Published:** 2021-06-26

**Authors:** Sarah Byford, Hristina Petkova, Barbara Barrett, Tamsin Ford, Dasha Nicholls, Mima Simic, Simon Gowers, Geraldine Macdonald, Ruth Stuart, Nuala Livingstone, Grace Kelly, Jonathan Kelly, Kandarp Joshi, Helen Smith, Ivan Eisler

**Affiliations:** 1 Institute of Psychiatry, Psychology & Neuroscience at King’s College London, PO24 David Goldberg Centre, De Crespigny Park, London, SE5 8AF UK; 2 Department of Psychiatry, University of Cambridge, Level 5 Clifford Allbutt Building, Cambridge Biomedical Campus, Hills Road, Cambridge, CB2 0AH UK; 3 Imperial College London, Division of Psychiatry, Department of Brain Sciences, Commonwealth Building, Du Cane Road, London, W12 0NN UK; 4 South London and Maudsley NHS Foundation Trust, Michael Rutter Centre for Children and Young People, De Crespigny Park, London, SE5 8AZ UK; 5 University of Liverpool, Mount Pleasant, Liverpool, L69 3BX UK; 6 University of Bristol, School for Policy Studies, 8 Priory Road, Bristol, BS8 1TZ UK; 7 Queen’s University Belfast, School of Social Sciences, Education & Social Work, 6 College Park Ave, Belfast, BT7 1PS UK; 8 Queen’s University Belfast, University Road, Belfast, BT7 1NN UK; 9 Beat, Unit 1 Chalk Hill House, 19 Rosary Road, Norwich, Norfolk NR1 1SZ UK; 10 NHS Grampian and University of Aberdeen, CAMHS, City Hospital, Park Road, Aberdeen, AB24 5AU UK; 11 NHS Ayrshire and Arran, South CAMHS/NSAIU, CAMHS, Arrol Park Resource Centre, House 1, Doonfoot Road, Ayr, KA7 2DW UK; 12 South London and Maudsley NHS Foundation Trust, Maudsley Centre for Child and Adolescent Eating Disorders, Michael Rutter Centre for Children and Young People, De Crespigny Park, London, SE5 8AZ UK

**Keywords:** Anorexia nervosa, Cost-effectiveness analysis, Specialist eating disorders services, Child and adolescent mental health services

## Abstract

**Background:**

Evidence suggests specialist eating disorders services for children and adolescents with anorexia nervosa have the potential to improve outcomes and reduce costs through reduced hospital admissions. This study aimed to evaluate the cost-effectiveness of assessment and diagnosis in community-based specialist child and adolescent mental health services (CAMHS) compared to generic CAMHS for children and adolescents with anorexia nervosa.

**Method:**

Observational, surveillance study of children and adolescents aged 8 to 17, in contact with community-based CAMHS in the UK or Republic of Ireland for a first episode of anorexia nervosa. Data were reported by clinicians at baseline, 6 and 12-months follow-up. Outcomes included the Children’s Global Assessment Scale (CGAS) and percentage of median expected body mass for age and sex (%mBMI). Service use data included paediatric and psychiatric inpatient admissions, outpatient and day-patient attendances. A joint distribution of incremental mean costs and effects for each group was generated using bootstrapping to explore the probability that each service is the optimal choice, subject to a range of values a decision-maker might be willing to pay for outcome improvements. Uncertainty was explored using cost-effectiveness acceptability curves.

**Results:**

Two hundred ninety-eight children and adolescents met inclusion criteria. At 12-month follow-up, there were no significant differences in total costs or outcomes between specialist eating disorders services and generic CAMHS. However, adjustment for pre-specified baseline covariates resulted in observed differences favouring specialist services, due to significantly poorer clinical status of the specialist group at baseline. Cost-effectiveness analysis using CGAS suggests that the probability of assessment in a specialist service being cost-effective compared to generic CAMHS ranges from 90 to 50%, dependent on willingness to pay for improvements in outcome.

**Conclusions:**

Assessment in a specialist eating disorders service for children and adolescents with anorexia nervosa may have a higher probability of being cost-effective than assessment in generic CAMHS.

**Trial registration:**

ISRCTN12676087. Date of registration 07/01/2014.

## Plain English summary

Specialist eating disorders services may improve outcomes and reduce hospitalisations for children and adolescents with anorexia nervosa. Reductions in hospitalisation could save money for the NHS and are better for young people because hospitalisation disrupts their home life, social life and education. This study evaluated outcomes and costs of specialist eating disorders services compared to general child and adolescent mental health services (CAMHS) for children and adolescents with anorexia nervosa.

Children and adolescents were identified by contacting child and adolescent psychiatrists in the UK and Ireland and asking them to report any new cases of anorexia nervosa. These psychiatrists identified 298 young people aged 8 to 17 with an anorexia nervosa diagnosis for the first time. The psychiatrists provided information on the health services these young people used and how they were doing when they were first diagnosed and 6 months and 1 year later.

Children and adolescents in specialist services were more severely ill than those in CAMHS when they were first diagnosed. Despite this, care for the young people in specialist services cost about the same as for those diagnosed in CAMHS, and their outcomes after 1 year were similar. This work showed that specialist services may be better value for money than CAMHS.

## Background

Anorexia nervosa is an eating disorder associated with severe physical and psychological impairments and a significant cost burden [1–4]. Due to the life-threatening nature of the disorder, a substantial proportion of children and adolescents are admitted to hospital with evidence to suggest that the number of admissions is rising [5, 6]. Admissions are disruptive to family, school and social life, costly to health services and relapse rates following inpatient treatment are high [7, 8]. In the United Kingdom (UK) and Republic of Ireland, children and adolescents with anorexia nervosa are commonly assessed and diagnosed by generic child and adolescent mental health services (CAMHS) or specialist community-based eating disorders services. Few studies have compared the relative benefits of these different models of care but the available evidence suggests specialist outpatient treatment may be more effective than generic CAMHS [9, 10]. Economic evidence also supports the case for specialist services, suggesting that specialist outpatient treatment is cost-effective compared to both inpatient treatment and generic outpatient treatment [11]. However, available data are over 10 years old and service configurations may now be very different. No other economic evaluations of specialist eating disorders services for children and adolescents were identified. A recent systematic review found only nine economic evaluations of treatment interventions for all eating disorders in all ages [12], including the study referred to above [11]. Of the remainder, seven were carried out in adults or populations 16 years of age and over and age was unclear in one study. If the findings from the earlier study [11] can be shown to be generalisable, investing in specialist eating disorders services could have significant implications for the National Health Service (NHS), with the potential to improve health outcomes and the quality of life of young people and their families, and reduce costs through reduced admissions.

## Methods

### Aim

The Cost-effectiveness of models of care for young people with Eating Disorders (CostED) study aimed to evaluate the cost-effectiveness of assessment and diagnosis in community-based specialist CAMHS (e.g. specialist eating disorders services) and generic CAMHS for child and adolescent anorexia nervosa. The work presented here is a condensed version of one element of the CostED study, the full details of which are published elsewhere [13].

### Design

An observational, surveillance study was undertaken using the Child and Adolescent Psychiatry Surveillance System (CAPSS), a system designed to identify cases of rare childhood mental health conditions through monthly reporting by hospital, community and university-based child and adolescent psychiatrists in the UK and Republic of Ireland [14]. The CostED study involved monthly reporting of new cases of anorexia nervosa for a period of 8 months from 1st February to 30th September 2015.

### Setting

Community-based secondary or tertiary child and adolescent mental health services in the UK and Republic of Ireland.

### Procedures

All CAPSS clinicians were sent a study-specific protocol card and reporting instructions for new cases of anorexia nervosa and asked to check boxes to confirm any new cases of anorexia nervosa seen in the preceding month, or to check a “nil return” box if appropriate. The protocol card detailed the case notification definition for DSM5 anorexia nervosa [15] (see Appendix) which aimed to guide clinicians in their decision of whether to report a positive case. A tear-off slip was provided to enable psychiatrists to keep a record of the patients they reported. Positive returns were allocated a unique CAPSS ID number by the CAPSS administrator and notified to the CostED trial manager, who contacted the reporting clinician directly to request completion of a baseline questionnaire.

### Participants

Cases were eligible for inclusion if the young person: (1) was aged 8 to 17 years; (2) had no previous episode of anorexia nervosa that had come to the attention of services; (3) received a clinical assessment in the reporting service during the study surveillance period; (4) had not been referred from another secondary health service, to ensure assessment and diagnosis had not happened prior to the surveillance period; (5) were notified by a community-based service; and (6) had the following clinical symptoms reported: “Restriction of energy intake relative to requirements” and “Persistent behaviour that interferes with weight gain, despite low weight”. These two symptoms were used to provide an initial assessment of case eligibility and were subsequently checked using a tighter DSM5 analytic definition (see Appendix). Only one case meeting the broad criteria failed to meet the tighter criteria, thus confirming the validity of the broad criteria applied. Cases were excluded if the data were insufficient to assess eligibility.

### Data

Data were collected at baseline using questionnaires sent to clinicians notifying a positive case of anorexia nervosa and, if the case was found to be eligible for inclusion, follow-up questionnaires were sent 6 and 12-months after the date the case was assessed and diagnosed, as reported by clinicians in the baseline questionnaires. Clinicians completed all questionnaires from clinical records.

In line with CAPSS procedures and ethics requirements, baseline questionnaires contained a limited set of patient identifiers to describe the sample and identify duplicate notifications. Patient identifiers included NHS or Community Health Index number (unique patient identifiers used in the included regions), hospital number, first half of postcode or town of residence for Republic of Ireland, gender, date of birth and ethnicity (White, Mixed, Asian, Black, Chinese, Other or Unknown). In Northern Ireland, identifiers were further limited to age in years and months (instead of date of birth) and hospital identifier (instead of hospital number), to further reduce the risk of patient identification given the small geographic area. In keeping with the requirements of the Northern Ireland Privacy Advisory Committee, all patient identifiable data from Northern Ireland were retained by the local research team, de-duplicated, anonymised and subsequently sent for analysis to the central research team in King’s College London.

The management of duplicates was dependent on the outcome for the original notification for which a duplicate had been identified. Four scenarios were considered, and each was assessed in different ways, as follows:

- Duplicates of notifications where the first notification met the study inclusion criteria, were excluded and the original notification retained.
- Duplicates of notifications where the first notification had been excluded due to age (too young) or clinical ineligibility, were assessed as a new case to determine if the case now met eligibility criteria.
- Duplicates of notifications where the first notification had been excluded due to a previous episode of anorexia nervosa, an assessment and diagnosis date prior to the study recruitment period, or referral from another secondary care service were excluded.
- Duplicates of notifications where the first notification contained insufficient information to judge eligibility for inclusion (for example, missing date of birth), were checked to see if the second notification contained the information missing from the first and, if available, the first notification was reassessed for eligibility and the duplicate excluded.

Baseline questionnaires covered characteristics of the notifying service (to enable classification of services as specialist or generic), clinical characteristics of the notified case and outcome measures (to assess eligibility and provide baseline assessments of outcome), and referral pathway information (to ensure assessment and diagnosis had not happened prior to the surveillance period and to determine whether the case had been referred to another service). Follow-up questionnaires included characteristics of the service, clinical characteristics of the notified case, outcome measures, and use of health services.

Incomplete or unreturned questionnaires were pursued via email, post and telephone. Cases where any symptoms required for case definition remained absent despite chasing, were assessed for eligibility by a consultant child and adolescent psychiatrist (MS). Those determined by MS to have too much data missing to assess, were excluded.

### Clinical characteristics and outcome measures

Clinical characteristics included weight and height and a range of symptoms to support diagnosis (see Appendix). Weight and height were used to calculate percentage of median expected body mass index (BMI) for age and sex (%mBMI) [16, 17]. In addition, clinicians were asked to report scores for two generic outcome measures: the Children’s Global Assessment Scale (CGAS) [18] and the Health of the Nation Outcome Score for Children and Adolescents (HoNOSCA) [19]. The CGAS is completed by clinicians and used to rate emotional and behavioural functioning of young people in the family, school, and social context. Clinicians score the young person on a scale from 1 to 100 using a classification which includes ten categories ranging from ‘Extremely impaired’ (score 1–10) to ‘Doing very well’ (score 91–100) [18]. All CostED questionnaires contained a copy of the CGAS classification system, describing each of the ten categories, to support scoring by clinicians. The HoNOSCA is a routine outcome measurement tool rating 13 clinical features on a 5-point severity scale. It assesses behaviours, impairments, symptoms, and social functioning of young people with mental health problems, producing a total score on a scale from 0 to 52, where a higher score indicates a poorer outcome.

Although there is a preference for generic measures of health-related quality of life capable of generating quality adjusted life years (QALYs) in economic evaluations – since this allows for comparison across populations and disorders when making resource allocation decisions – no such measure is routinely collected by NHS clinical services. Given the reliance on data from clinical records, it was therefore not possible to undertake a cost-utility analysis, which uses QALYs as the measure of effect, as is preferred, for example, by the National Institute for Health and Care Excellence which produces clinical guidelines in England [20].

### Service use and costs

Since data were collected from clinical records, the perspective of the economic evaluation was limited to health services for which data were likely to be available to clinicians. This included: hospital inpatient admissions (including paediatric, general child/adolescent or adult psychiatry, child/adolescent or adult eating disorders unit, or other), out-patient attendances (including paediatrics, specialist eating disorders service, and other psychiatric service) and day-patient attendances (including paediatrics, specialist eating disorders service, and other psychiatric service). Adult services were included because some adolescents may have transferred to adult services by follow-up.

All costs, in pounds sterling, were for the 2015/16 financial year, in line with the data collection period. Discounting was not necessary as follow-up was not longer than 12-months. Costs for NHS hospital admissions and outpatient and day-patient contacts, including CAMHS contacts, were taken from NHS reference costs [21]. Independent sector costs were provided by a range of independent sector organisations and NHS Trusts via personal communications. Unit costs are summarised in Table 1.

**Table 1:** Unit costs for health services used

Service	Unit cost	Source
NHS inpatient cost per night		
Eating disorders unit – child/adolescent	510.14	NHS Reference Costs^a^
Eating disorders unit – adult	455.02	NHS Reference Costs^a^
General psychiatry – child/adolescent	633.07	NHS Reference Costs^a^
General psychiatry – adult	197.29	NHS Reference Costs^a^
Paediatric if stay = 1 night	426.99	NHS Reference Costs^a^
Paediatric if stay> 1 night	592.27	NHS Reference Costs^a^
Other NHS	389.10	NHS Reference Costs^a^
Independent sector inpatient cost per night		
Eating disorders unit – child/adolescent	695.00	Personal communication
General psychiatry – child/adolescent	668.00	Personal communication
Outpatient cost per contact		
Eating disorders service	262.12	NHS Reference Costs^a^
Other psychiatry	298.57	NHS Reference Costs^a^
Paediatric	194.36	NHS Reference Costs^a^
Day-patient cost per contact		
Eating disorders service	274.21	NHS Reference Costs^a^
Other psychiatry	326.16	NHS Reference Costs^a^
Paediatric	446.60	NHS Reference Costs^a^

^a^Department of Health. NHS reference costs. 2015

### Classification of services

Specialist eating disorders services are not clearly defined, with definitions changing over time [22, 23]. In order to classify services as specialist or generic, an online Delphi survey was undertaken to obtain consensus on the key features of a specialist child and adolescent eating disorders service from a range of stakeholders, including service users and their families, child and adolescent psychiatrists, paediatricians, other eating disorders professionals and service commissioners [13]. Further information regarding the characteristics included in the Delphi survey can be found in the published report [13]. Characteristics meeting pre-defined consensus thresholds to be considered important for the classification of services as specialist eating disorders services included: offering specialist outpatient treatment for eating disorders, providing multi-disciplinary specialist outpatient clinics dedicated to eating disorders and holding weekly multi-disciplinary meetings dedicated to eating disorders. All cases notified by a service meeting all three criteria were classified as specialist; all other services were classified as generic CAMHS.

### Data analysis

Analyses compared participants assessed in specialist eating disorders services with those assessed in generic CAMHS. All analyses were adjusted for pre-specified baseline covariates including CGAS, %mBMI, age, sex and region (England, Wales, Scotland, Northern Ireland, Republic of Ireland). Outcomes and total costs per participant were compared using standard parametric t-tests [24], with the robustness of this approach for costs confirmed using bootstrapping (5000 iterations) [25]. Analyses used complete case data with the impact of missing cost and outcome data over the follow-up period tested in sensitivity analyses using multiple imputations [26]. Alternative approaches to the handling of observational data were considered, such as matching and stratification [27], but small sample sizes, compounded by missing data at baseline and follow-up, and imbalance in the size of the two groups (specialist versus generic) precluded these, and more sophisticated, approaches.

The pre-specified primary measure of effectiveness, as outlined in the original protocol, was the HoNOSCA. The %mBMI was specified as a secondary cost-effectiveness analysis because, although more narrowly focused, data on weight and height were expected to be available for a greater proportion of the sample. From the baseline questionnaires, however, the level of missing HoNOSCA data was substantial (79% missing at baseline) and the protocol was amended replacing the HoNOSCA with the CGAS, for which a greater proportion of data were available (8% missing at baseline).

Cost-effectiveness was explored in a decision-making context, focusing on the probability of one service type being cost-effective compared to the other, given the data available, rather than a focus on statistical significance. This is the recommended approach for economic evaluation in the UK [28]. Cost-effectiveness was assessed using incremental cost-effectiveness ratios (difference in mean cost between two interventions divided by the difference in mean effect) and taking the recommended net benefit approach [29]. Incremental cost-effectiveness ratios were calculated for scenarios where one intervention demonstrates higher costs and better outcomes (it is unnecessary to calculate these ratios where one group shows both lower costs and better outcomes as it ‘dominates’ the other group). A joint distribution of incremental mean costs and effects for each group was generated using bootstrapping [25] to explore the probability that each service is the optimal choice, subject to a range of values a decision-maker might be willing to pay for outcome improvements (£0 to £30,000). Cost-effectiveness acceptability curves were generated by plotting these probabilities for a range of possible values of the ceiling ratio [30, 31]. These curves are the recommended approach to dealing with uncertainty around the cost and effect estimates and the maximum cost-effectiveness ratio that decision-maker would consider acceptable [28, 31].

## Results

### Sample

Over the eight-month surveillance period, 6401 case notification cards were sent to clinicians and 3211 were returned (50.16%). Of these, 997 positive cases of anorexia nervosa were reported and 2214 were nil returns. Of the 997 positive returns, 48 (5%) were excluded due to reporting errors or clinicans stating that they did not wish to take part in the study (due to lack of time, retirement etc.). Baseline questionnaires were sent to clinicians who reported the remaining 949 positive returns, and 597 (63%) were returned. Of these, 299 (49%) were ineligible (due to age, previous episode of anorexia nervosa, date of assessment outside the surveillance period, referral from another secondary care service, current inpatient, duplicate notification or insufficient information to assess diagnosis). Thus 298 cases were included in the study, 192 (64%) assessed in a specialist eating disorders service and 106 (36%) assessed in generic CAMHS. Clinicians completed and returned follow-up questionnaires for 220 cases at 6-months (74%), 147 specialist (77%) and 73 generic (69%), and 187 cases at 12-months (63%), 137 specialist (71%) and 50 generic (47%). Included cases were referred from a total of 79 services, 50 of which were generic CAMHS (63%) and 29 were specialist eating disorders services (37%).

Demographic and baseline characteristics are reported in Table 2. Mean age (approx. 15 years), proportion female (92%) and ethnicity (92–94% Any White background) were similar between the two groups and in line with evidence of the demographic at risk for anorexia nervosa [32]. Baseline clinical variables suggest the sample were significantly impaired. Mean %mBMI was approximately 83%, falling within the range expected for a diagnosis of anorexia nervosa (< 85%). Mean CGAS score was approximately 44, falling within the ‘obvious problems’ range [18], and mean total HoNOSCA score was approximately 19, indicative of a severity similar to that at inpatient admission [9, 33]. Comparing the two groups, %mBMI was similar in the two groups (82.7% versus 83.6%) but differences were evident for CGAS (mean 43 specialist versus 48 generic) and HoNOSCA (mean 21 specialist versus 15 generic).

**Table 2:** Baseline characteristics of specialist versus generic cases

	Specialist		Generic	
	N	Mean (SD) or %	N	Mean (SD) or %
Age	192	15.09 (1.60)	106	14.84 (1.66)
Sex				
Female	176	91.67%	97	91.51%
Male	16	8.33%	9	8.49%
Ethnicity				
Any White	174	91.58%	95	94.06%
Other	16	8.42%	6	5.94%
Clinical status				
%mBMI	191	82.70 (11.11)	105	83.60 (9.90)
CGAS	174	43.22 (14.40)	99	47.86 (13.29)
HoNOSCA	45	21.04 (8.43)	16	14.88 (5.77)

NB: Not all percentages add up to 100% due to rounding

### Service use

Mean use of inpatient, outpatient and day-patient services over the 12-month follow-up is reported in Table 3. Mean number of inpatient admissions and inpatient nights per participant were similar in the two groups, although participants assessed in specialist services spent longer, on average, in eating disorders facilities (20 versus 13 nights) and less time on general psychiatry wards (7 versus 13 nights). The pattern for outpatient attendances was similar, with participants assessed in specialist services having more contacts in eating disorders facilities (27 versus 15 attendances) but fewer general psychiatry contacts (3 versus 12 attendances) and a similar number of contacts in total (30 versus 27 attendances). Day-patient services were accessed by 11% of the specialist group but only 4% of the generic group, with average number of attendances also being higher for the specialist group (5 versus 1 attendance). Most of these contacts were in eating disorders services.

**Table 3:** Service use between baseline and 12-month follow-up

	Specialist (*n* = 137)		Generic (*n* = 50)	
Service	Mean (SD)	% using	Mean (SD)	% using
Inpatient admissions	0.54 (1.06)	28.47%	0.60 (1.20)	30.00%
Inpatient nights	31.75 (80.03)	28.47%	30.78 (68.65)	30.00%
Paediatric – NHS	2.18 (9.28)	15.33%	4.72 (12.96)	20.00%
Eating disorders – NHS	5.56 (37.72)	2.92%	0.28 (1.98)	2.00%
Eating disorders – Independent	14.28 (57.93)	8.76%	12.84 (49.89)	8.00%
Psychiatry – NHS	5.38 (24.47)	6.57%	11.58 (39.42)	12.00%
Psychiatry – Independent	1.39 (12.43)	2.19%	1.36 (9.62)	2.00%
Other – NHS	0.53 (6.15)	1.46%	0.00 (0.00)	0.00%
Outpatient attendances	29.98 (17.70)	98.54%	27.14 (32.62)	96.00%
Paediatric	0.07 (0.34)	5.11%	0.98 (2.46)	28.00%
Eating disorders	27.11 (18.11)	92.70%	14.58 (32.07)	56.00%
Psychiatry	2.80 (8.94)	17.52%	11.58 (13.55)	68.00%
Day patient attendances	4.61 (16.60)	10.95%	0.86 (5.66)	4.00%
Paediatric	0.00 (0.00)	0.00%	0.06 (0.42)	2.00%
Eating disorders	4.25 (16.13)	10.22%	0.80 (5.66)	2.00%
Psychiatry	0.37 (4.27)	0.73%	0.00 (0.00)	0.00%

### Cost

Cost results are reported in Table 4. Although there were only small observed differences in total costs, adjustment for pre-specified baseline variables made a substantial difference due to baseline differences, particularly in the CGAS. Adjusted analyses suggested large differences in favour of specialist services (costs lower on average), although these differences were not significant. Imputation of missing data made little difference to the results, with costs remaining lower in the specialist group in adjusted analyses.

**Table 4:** Total cost per participant between baseline and 12-month follow-up

	Specialist	Generic	Unadjusted^a^		Adjusted^ab^	
Service	Mean £ (SD)	Mean £ (SD)	Mean difference (95% CI)	*p*-value	Mean difference (95% CI)	*p*-value
Baseline to 6 months	*n* = 147	*n* = 73				
Inpatient	11105 (28877)	11179 (29050)				
Outpatient	4764 (2866)	4310 (3975)				
Day-patient	947 (3468)	299 (1708)				
Total	16817 (28469)	15789 (28184)	1028 (− 6979 to 9036)	0.801	− 3586 (−11999 to 4827)	0.402
Six to 12 months	*n* = 137	*n* = 50				
Inpatient	8153 (24763)	8224 (20894)				
Outpatient	3103 (3058)	3279 (5775)				
Day-patient	306 (1703)	44 (310)				
Total	11562 (24905)	11547 (22133)	14 (− 7875 to 7903)	0.997	− 2785 (− 11241 to 5670)	0.516
Baseline to 12 months	*n* = 137	*n* = 50				
Inpatient	19462 (49946)	19755 (44677)				
Outpatient	7955 (4722)	7470 (8499)				
Day-patient	1284 (4608)	246 (1559)				
Total	28700 (49716)	27471 (44317)	1230 (−14529 to 16988)	0.878	−7106 (−23590 to 9379)	0.396

NB: Not all totals add up due to rounding; ^a^Standard parametric tests with validity tested using bootstrapping (bootstrapped results similar so not reported); ^b^Adjusted for baseline CGAS, baseline %mBMI, age, sex and region

### Outcomes

Outcome results are reported in Table 5. At baseline, %mBMI was not statistically different between the two groups. However, both CGAS and HoNOSCA scores were significantly worse in the specialist group. At the 6-month follow-up, in adjusted analyses, %mBMI was significantly higher in the specialist than the generic group. Although %mBMI remained higher for the specialist group at 12-months, the difference was no longer significant. No significant differences were identified for CGAS or HoNOSCA at either follow-up. Imputation of missing data made little difference, with imputed results being similar to complete case results.

**Table 5:** Outcome measures at baseline, 6 and 12-month follow-up

	Specialist		Generic		Unadjusted^a^		Adjusted^ab^	
Outcome measure	N	Mean (SD)	n	Mean (SD)	Mean difference (95% CI)	*p*-value	Mean difference (95% CI)	*p*-value
Baseline								
%mBMI	191	82.70 (11.10)	105	83.60 (9.90)	−0.90 (−3.46 to 1.66)	0.489		
CGAS	174	43.22 (14.40)	99	47.86 (13.29)	−4.64 (−8.11 to −1.17)	**0.009**		
HoNOSCA	45	21.04 (8.43)	16	14.88 (5.77)	6.17 (1.60 to 10.74)	**0.009**		
6-months								
%mBMI	143	91.98 (8.51)	67	89.62 (12.89)	2.37 (−0.59 to 5.31)	0.116	2.58 (0.16 to 5.01)	**0.037**
CGAS	115	58.94 (17.17)	55	63.27 (17.05)	−4.33 (−9.88 to 1.21)	0.125	0.49 (−5.14 to 6.12)	0.864
HoNOSCA	17	9.47 (7.43)	16	11.88 (10.31)	−2.40 (−8.76 to 3.95)	0.446	−6.61 (−15.54 to 2.31)	0.140
12-months								
%mBMI	106	94.70 (10.61)	39	93.36 (9.46)	1.34 (−2.48 to 5.16)	0.489	0.09 (−3.54 to 3.73)	0.960
CGAS	97	68.39 (17.95)	38	71.58 (21.41)	−3.19 (−10.37 to 4.00)	0.382	−0.65 (−8.26 to 6.96)	0.866
HoNOSCA	12	7.42 (4.48)	7	13.57 (16.94)	−6.15 (−16.88 to 4.57)	0.243	−12.42 (−31.07 to 6.23)	0.171

^a^Standard parametric tests; ^b^Adjusted for baseline CGAS, baseline %mBMI, age, sex and region; *%mBMI* percentage of median expected BMI for age and sex (higher percentage, better outcome), *CGAS* Children’s Global Assessment Scale (scored between 1 and 100; higher score, better outcome), *HoNOSCA* Health of the Nation Outcome Score for Children and Adolescents (scored between 0 and 52; higher score, poorer outcome)

### Cost-effectiveness

**Fig. 1:**
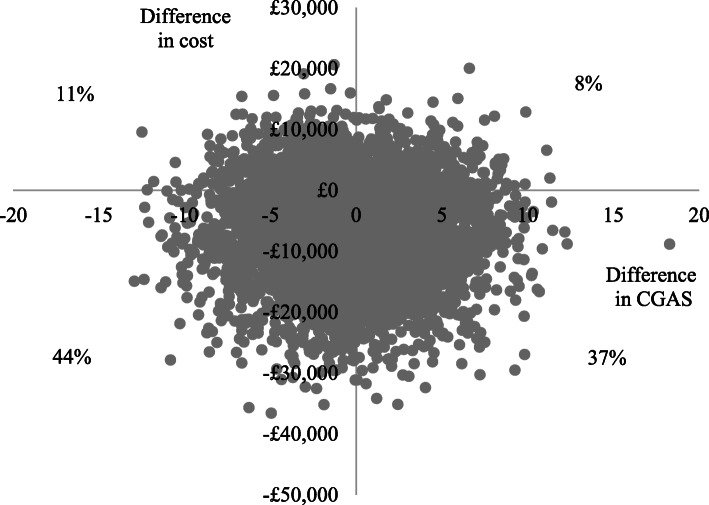
Cost-effectiveness plane for CGAS at 12-months

**Fig. 2:**
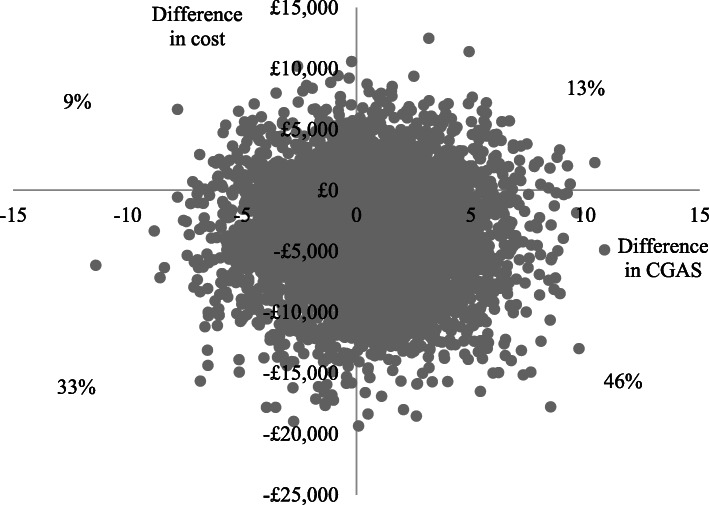
Cost-effectiveness plane for CGAS at 6-months

Using the CGAS (primary measure of effect) at 12-months (primary endpoint), adjusted total costs per participant were lower and adjusted CGAS scores slightly lower (poorer outcomes) in the specialist than the generic group, generating an incremental cost-effectiveness ratio of £10,932 (−£7106/− 0.65). This suggests that assessment in a generic service generates a unit improvement in CGAS score for an additional cost of approximately £11,000, compared to specialist services. The cost-effectiveness plane in Fig. 1 illustrates that the scatter points fall in all four quadrants, but the largest proportion are in the South-West quadrant where specialist services are cheaper (below the x-axis) and less effective (left of the y-axis). At the 6-month follow-up, adjusted total costs per participant were lower in the specialist group and adjusted CGAS scores slightly higher (better outcomes), so specialist services dominated generic services (Fig. 2).

**Fig. 3:**
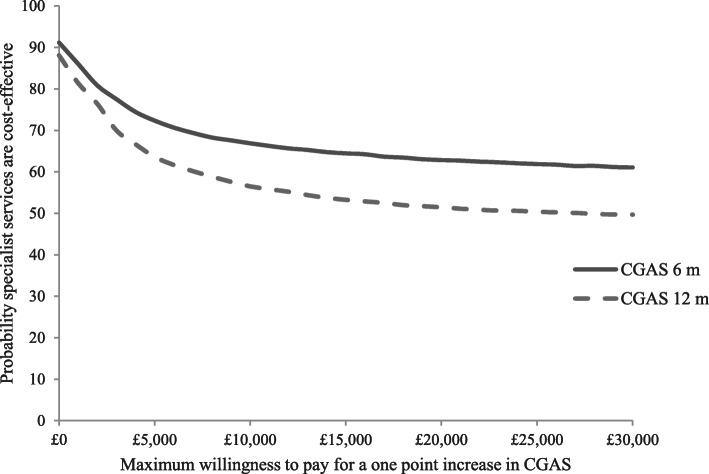
Cost-effectiveness acceptability curves for CGAS at 6 and 12-months

Cost-effectiveness acceptability curves (Fig. 3), are downward sloping, suggesting that the cost-effectiveness of specialist eating disorders services declines as willingness to pay for improvements in CGAS increase, but does not fall below 50% over the willingness to pay range tested. This is due to the lower cost of the specialist group and the small differences in effects (i.e., at low levels of willingness to pay, society favours the cheaper option, but at higher levels of willingness to pay, society becomes increasingly indifferent between the two options). The curves illustrate that there is a higher probability of assessment in specialist services being cost-effective compared to generic services at both follow-ups, with probabilities at 12-months ranging from around 90% at zero willingness to pay for improvements in CGAS to 50% at willingness to pay of £30,000.

**Fig. 4:**
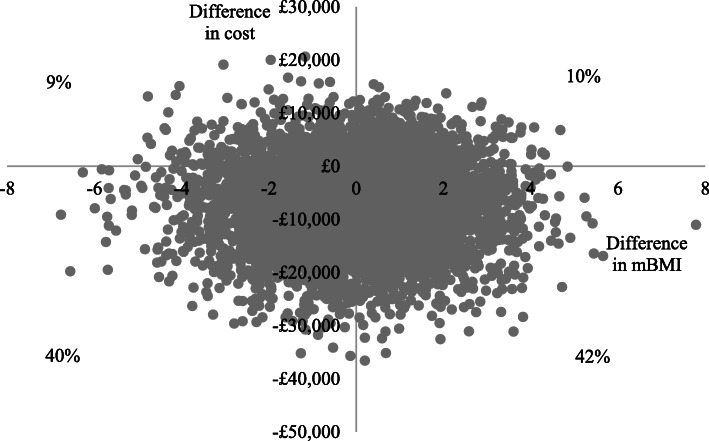
Cost-effectiveness plane for %mBMI at 12-months

**Fig. 5:**
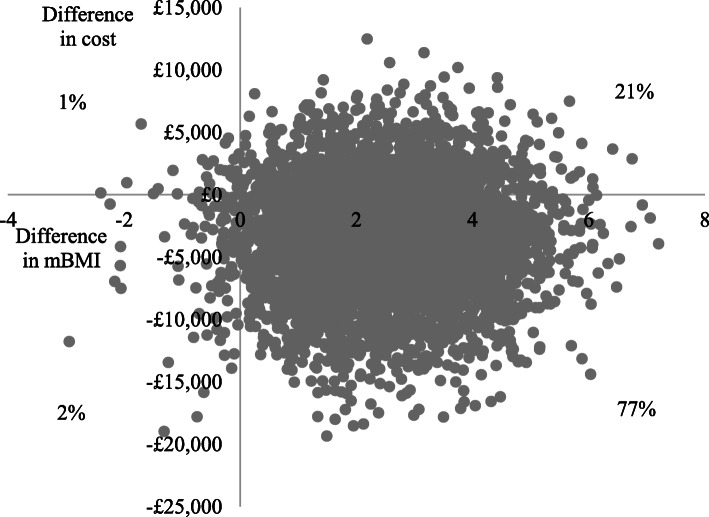
Cost-effectiveness plane for %mBMI at 6-months

**Fig. 6:**
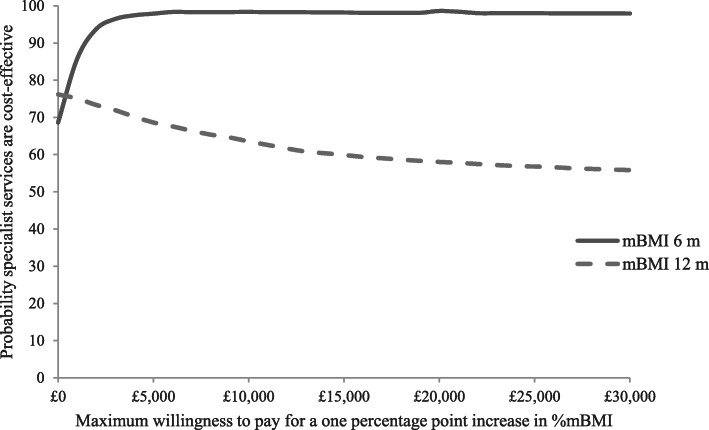
Cost-effectiveness acceptability curve for %mBMI at 6 and 12-months

In terms of %mBMI, the secondary measure of effect, adjusted total costs per participant were lower and %mBMI scores were slightly higher (better outcome) in the specialist than the generic group at both the 6-month and 12-month follow-ups, so specialist services dominated generic services (Figs. 4 and 5). Cost-effectiveness acceptability curves (Fig. 6) suggest there is a higher probability of initial assessment in specialist services being cost-effective compared to generic services at both follow-ups, with probabilities at 12-months ranging from 76% at zero willingness to pay to 56% at willingness to pay of £30,000.

## Discussion

### Overview of the results

The results presented suggest that for children and adolescents aged 8 to 17 with anorexia nervosa in the UK and Republic of Ireland, assessment in specialist eating disorders services has a similar or higher probability of being cost-effective than assessment in generic CAMHS. At 12-months follow-up, specialist eating disorders services had a higher probability of being cost-effective than generic CAMHS at low levels of willingness to pay for improvements in CGAS score and improvements in %mBMI; at higher levels of willingness to pay, the probability of either specialist or generic being cost-effective was around 50%. At 6-months follow-up, assessment in specialist eating disorders services had a higher probability of being cost-effective for the full range of willingness to pay values tested (£0 to £30,000). Without knowledge of the amount society is willing to pay for improvements in CGAS or %mBMI, no firm conclusions can be reached. However, we would hypothesise that it is unlikely that decision-makers would be willing to pay in excess of £30,000 for a one percentage point improvement in either measure, which is the minimum amount that would need to be spent for generic CAMHS to have a similar or higher probability of being cost-effective compared to specialist services.

Cost-effectiveness findings in favour of specialist services were due to similar outcomes in the two groups alongside lower costs in the specialist group, following adjustment for poorer baseline clinical status in the specialist group. Despite this poorer baseline status, children and adolescents assessed in specialist services achieved significantly better weight outcomes 6-months after assessment and similar outcomes at 12-months compare to those assessed in generic CAMHS, with a similar level of health service use, and thus similar costs.

These results are in line with previous evidence to support the cost-effectiveness of specialist services for children and adolescents with anorexia nervosa. One RCT carried out in the UK, concluded that specialist out-patient treatment has a higher probability of being cost-effective than inpatient or general outpatient treatment [9, 11]. In common with the CostED study, there were no differences in outcomes, however the specialist outpatient group had substantially lower costs than the comparison groups over two-year follow-up. Similar results were seen in a London-based study [34]. Whilst no significant differences in outcome were evident, admissions were significantly lower over one-year follow-up for children and adolescents assessed and treated in a specialist service (15% admitted) compared with those assessed and treated in a non-specialist service (40%).

From a pragmatic point of view, combining the CostED study results with previous evidence to support the cost-effectiveness of specialist community-based services [11] and to suggest that specialist services reduce admissions and costs [34], the evidence as a whole supports the provision of specialist eating disorders services, which is in line with recent guidance for and investment into the development of community eating disorders services for children and adolescents in England [22]. In addition, the CostED results should be considered alongside other factors of relevance to investment decisions, in particular the preferences of patients and carers. Existing evidence suggests a preference from these groups for specialist services [34–36], making the differential use of inpatient and outpatient services identified – with children and adolescents assessed in specialist services having greater contact with eating disorders facilities and less contact with general psychiatry services than those assessed in generic services – of particular importance.

### Strengths and limitations

Anorexia nervosa is a relatively rare disorder, in comparison to other childhood mental health disorders, such as depression, making it difficult to recruit adequately powered samples for clinical trials. This surveillance study was able to gather case notifications from almost one hundred services across the UK and Republic of Ireland, which for a clinical trial would be prohibitively expensive. After accounting for duplicates and withdrawals, 298 cases were eligible for inclusion, a sample which is larger than RCTs in this population have been able to achieve to date [9, 37, 38]. However, it should be noted that this sample does not reflect the entire population of children and adolescents with an anorexia nervosa diagnosis for the first time in this age range, as only 50% of case notification cards were returned. Whilst a proportion of these would have been ‘nil’ returns (no case of anorexia nervosa to report), some will have been missing cases of anorexia nervosa meeting the study inclusion criteria.

In addition, the study benefits from being ‘real world’, reflecting actual clinical practice with standard clinical populations, broad inclusion criteria and limited exclusion criteria. However, the results were clearly affected by baseline differences in outcome scores between the two groups, which is a limitation of evaluations that do not randomly allocate participants, and may also reflect a level of participant self-selection, at least in those participants with access to both types of services. All analyses were therefore adjusted for baseline variables to minimise the impact of this limitation. Alternative approaches to the handling of observational data were considered, but small sample sizes, missing data, and imbalance in the size of the two groups precluded such approaches and thus the results should be interpreted within the context of the methodological limitations of the study.

At baseline, 63% of questionnaires were returned allowing cases to be assessed for eligibility for inclusion in the follow-up. Of those assessed as eligible, 74% of 6-month follow-up questionnaires and 63% of 12-month follow-up questionnaires were returned. Missing data was therefore a limitation which impacts upon the generalisability and interpretation of the results. However, imputation of missing data produced very similar results to complete case analyses, giving greater confidence in the results presented. In contrast, missing items in returned questionnaires were small in number, except for the HoNOSCA, suggesting that clinical services are not using this measure in routine practice. As a result, the HoNOSCA was removed as the pre-specified primary outcome measure and replaced with the CGAS, which had substantially higher response rates.

Some limitations arose directly from reliance on retrospective data contained within clinical records, rather than prospective collection from children and adolescents. In particular, availability of outcome data was limited to CGAS scores and %mBMI. These outcomes are too narrow to capture all impacts clinical services may have on the quality of life of children and adolescents with anorexia nervosa which suggests the need for clinical services to consider the consistent collection of measures of change, including measures relevant to eating disorders and broader measures of quality of life, to aid investigation of the effectiveness of services from both a clinical and a research point of view. Similarly, service use was limited to those health services for which data were likely to be available to all clinicians from clinical records, although this did capture the key services found in previous evaluations to account for the vast majority (over 90%) of the total costs in similar samples [11, 34]. Adjustment for confounding factors was also limited by the availability of data in clinical records, and thus it is possible that unobserved factors of importance were missed.

Whilst recruitment from across the UK and Republic of Ireland avoids biases inherent in studying clinical samples from a small number of services across a narrow range of geographical locations, bias in reporting and questionnaire completion is likely to be a problem. Specialist services have larger numbers of eating disorders cases than generic CAMHS, placing a greater questionnaire completion burden on clinicians, which may reduce their willingness to respond. However, those in specialist services are more likely to have an interest in the research, which may have a positive effect on their willingness to take part. Whilst the direction of any bias at the reporting stage is unknown, it is worth noting that a much larger proportion of the total number of services reporting at least one notification were generic CAMHS (over 60%), suggesting that these services were engaging with the study. However, biases in questionnaire completion at follow-up were evident, with completion rates being higher in the specialist group (77% at 6-months; 71% at 12-months follow-up) compared to generic CAMHS (69% at 6-months; 47% at 12-months follow-up).

The CostED study was also open to bias from loss to follow-up due to referral elsewhere or discharge – likely to be a particular problem as adolescents reach 18 years of age and move to university or to take up employment. Follow-up data were less likely to be available for participants who were doing well and had been discharged prior to follow-up, for participants who were doing badly and had been admitted to hospital or referred from generic CAMHS to specialist services, and for participants who had moved out of area. Although the small differences in outcomes between the groups may suggest that loss to follow-up due to discharge may also be similar, evidence that children and adolescents treated in generic services are supported for longer than those in specialist services [34] would suggest that loss of data due to discharge may have been greater for specialist services.

In terms of loss of data due to hospital admission, specialist services will generally remain in regular contact with their patients while they are in hospital and will also continue outpatient treatment following discharge. Although practice is variable, this is less likely to be the case in generic services, so data loss as a result of referral may have been greater in generic services. It is also worth noting that there was considerably greater loss to follow-up in generic services, particularly at 12-months (53% versus 29%), and if this indicates a greater proportion of participants doing badly who are admitted to hospital or referred to specialist community services, this would have biased the results in favour of generic CAMHS. This type of loss to follow-up may also explain the surprisingly low lengths of admissions in the CostED sample compared to other studies [34, 39].

A further bias may arise from the exclusion of children and adolescents admitted directly to inpatient facilities without any contact with community-based services, which may be more likely in areas where community-based specialist eating disorders services are not available. This potentially introduces a bias in favour of generic CAMHS since these children and adolescents will be receiving considerably more expensive care as inpatients than the cost of treating them in community-based generic services would have been. Finally, it should be noted that nationally applicable unit costs were applied to most services, which increases the generalisability of the results to the national picture but reduces variability and the local applicability of the results.

## Conclusion

Assessment and diagnosis in a specialist eating disorders service for children and adolescents with anorexia nervosa may have a higher probability of being cost-effective than assessment and diagnosis in generic CAMHS, as specialist services were able to achieve larger gains in clinical effectiveness but without additional expenditure.

## Data Availability

As a result of the collection of confidential patient data without consent, and approval from the Health Research Authority (following advice from the Confidentiality Advisory Group) for data to be provided for the purposes of the specified activity only, the datasets generated and analysed during the current study cannot be made publicly available for other purposes. However, the CostED research group will consider requests for further analysis on a case by case basis, subject to appropriate ethical/HRA CAG approvals. All data enquiries should be submitted to the corresponding author for consideration in the first instance. Access to available anonymised data may be granted following review and if appropriate permissions are in place.

## Appendix

### Case notification definition

Please report any child/young person aged 8 to 17 years and 11 months inclusive, who meets the case notification definition criteria below for the first time in the last month. One bullet point criterion from each group below should be fulfilled.

Group A

- Restriction of food, low body weight, or
- Weight less than expected for age

Group B

- Fear of gaining weight, or
- Fear of becoming fat, or
- Behaviour that interferes with weight gain, for example excessive exercising, self-induced vomiting, use of laxatives and diuretics

Group C

- Body image disturbance, or
- Persistent lack of recognition of the seriousness of the current low body weight

Exclusions

- Patients who are not underweight
- Patients with bulimia nervosa, binge eating disorder, avoidant restrictive food intake disorder or other failure to thrive presentations

### DSM5 analytic definition

The tighter DSM5 analytic definition used to check the validity of the broad ‘two-symptom’ definition, included the following symptoms:

1. “Restriction of energy intake relative to requirements” and
2. “Intense fear of gaining weight or of becoming fat” or “Persistent behaviour that interferes with weight gain, despite low weight” and
3. “Perception that body shape/size is larger than it is” or “Preoccupation with body weight and shape” or “Lack of recognition of the seriousness of the current low body weight”

### Clinical symptoms contained in the CostED questionnaires to support DSM5 diagnosis

Clinical symptoms included in the baseline, 6 and 12-month follow-up questionnaires were used to support diagnosis of DSM5 anorexia nervosa and included the following features which required a response of yes, no, not known or not applicable: restriction of energy intake relative to requirements; intense fear of gaining weight or of becoming fat; persistent behaviour that interferes with weight gain, despite low weight; perception that body shape/size is larger than it is; preoccupation with body weight and shape; lack of recognition of the seriousness of the current low body weight; excessive exercise; self-induced vomiting (including frequency); laxative or diuretic abuse; and binge eating (including frequency). In addition, for females, clinicians were asked if the young person had reached menarche and, if yes, whether there was secondary amenorrhoea.

## Abbreviations

BMI: Body Mass Index
%mBMI: Percentage of median expected body mass for age and sex
CAMHS: Child and Adolescent Mental Health Services
CAPSS: Child and Adolescent Psychiatry Surveillance System
CGAS: Children’s Global Assessment Scale
CostED: Cost-effectiveness of models of care for young people with Eating Disorders
DSM5: Diagnostic and Statistical Manual of Mental Disorders Fifth Edition
HoNOSCA: Health of the Nation Outcome Score for Children and Adolescents
NHS: National Health Service
QALY: Quality Adjusted Life Year
UK: United Kingdom
